# Thyroid Extract for Backward Children

**Published:** 1898-03-12

**Authors:** 


### THYROID EXTRACT FOR BACKWARD CHILDREN.

The use of thyroid extract in the treatment of sporadic cretinism is of course well known, but Dr. Clement Dukes, physician to Rugby School, suggests its employment as an aid in the development of children who are merely backward. He relates a case in which, although the child looked younger than her age, she was well-formed and intelligent, ate, drank, and slept well, was very well nourished, took her part both at home and at school the same as other children, and was like the other members of her family, except that she was short in stature and small made, and had a pallor which neither arsenic or iron had ever remedied. After giving thyroid tablets (one of five grains once a day, and after a month twice a day) her pallor disappeared, she became much more brisk and lively, and started growing; in fact, she became a different child altogether. This is certainly a case worthy of note, an experiment worthy of repetition.

